# Control of the Autophagy Pathway in Osteoarthritis: Key Regulators, Therapeutic Targets and Therapeutic Strategies

**DOI:** 10.3390/ijms22052700

**Published:** 2021-03-07

**Authors:** Maria Teresa Valenti, Luca Dalle Carbonare, Donato Zipeto, Monica Mottes

**Affiliations:** 1 Department of Medicine, Section of Internal Medicine, University of Verona, 37134 Verona, Italy; mariateresa.valenti@univr.it; 2 Department of Neurosciences, Biomedicine and Movement Sciences, Section of Biology and Genetics, University of Verona, 37134 Verona, Italy; donato.zipeto@univr.it (D.Z.); monica.mottes@univr.it (M.M.)

**Keywords:** mesenchimal stem cells, chondrocytic commitment, autophagy, osteoarthritis, miRNAs

## Abstract

Autophagy is involved in different degenerative diseases and it may control epigenetic modifications, metabolic processes, stem cells differentiation as well as apoptosis. Autophagy plays a key role in maintaining the homeostasis of cartilage, the tissue produced by chondrocytes; its impairment has been associated to cartilage dysfunctions such as osteoarthritis (OA). Due to their location in a reduced oxygen context, both differentiating and mature chondrocytes are at risk of premature apoptosis, which can be prevented by autophagy. AutophagomiRNAs, which regulate the autophagic process, have been found differentially expressed in OA. AutophagomiRNAs, as well as other regulatory molecules, may also be useful as therapeutic targets. In this review, we describe and discuss the role of autophagy in OA, focusing mainly on the control of autophagomiRNAs in OA pathogenesis and their potential therapeutic applications.

## 1. Background

Chondrogenesis, the process by which cartilage is formed, occurs as a result of mesenchymal cell condensation and chondroprogenitor cell differentiation. SOX9 is the master transcription factor for MSCs differentiation into chondrocytes [1]. During development, chondrogenesis is subject to complex regulation by several interplaying factors such as fibroblast growth factor (FGF), transforming growth factor β (TGFβ), bone morphogenetic proteins (BMPs) and Wnt signaling pathways. Among regulatory factors, TGFβ plays a key role in development. TGFβ receptors are present in many cells, and an integrin-applied force is required to release TGF-β from its prodomain. Force application has been shown to occurs through the prodomain of TGF-β and through the β subunit of the integrin [2]. In the process termed endochondral ossification, chondrocytes undergo proliferation and terminal differentiation to hypertrophy and apoptosis, whereby hypertrophic cartilage is replaced by bone tissue. In mature articular cartilage, instead, chondrocytes are responsible for the production and homeostatic maintenance of the extracellular matrix (ECM) components.

Structural ECM components are: collagens (mainly type II collagen), proteoglycans, (including aggrecan, decorin, biglycan and fibromodulin) and noncollagenous proteins, such as cartilage oligomeric protein (COMP), cartilage matrix protein (CMP) and fibronectin. The primary matrix degrading enzymes involved in cartilage turnover are metalloproteinases (MMPs) and cathepsins (CTS). In healthy articular cartilage, balanced degradation and synthesis by chondrocytes ensure tissue homeostasis.

Autophagy plays a key role in the preservation of cartilage integrity [3]. Besides playing a crucial role in adaptive response to different stimuli, it is also required for intracellular quality control and is involved in removing and recycling misfolded proteins, damaged organelles or dysfunctional cell components [4].

Authophagy may be distinguished into (i) macroautophagy; (ii) microautophagy; (iii) chaperone mediated autophagy. Macroautophagy represents the prevalent form of autophagy in different cell types. It starts with a membrane formation, the phagophore, which expands to engulf the cellular cargo, generating the autophagosome. This latter structure matures through fusion with lysosomes. mTOR is a major player in autophagy and acts as a signaling control point downstream of growth factor receptor signaling, hypoxia, ATP levels and insulin signaling. It is activated downstream of Akt kinase, PI3-kinase and growth factor receptor and acts to inhibit autophagy by modulating the Ulk1 (Atg1) complex. In response to the autophagy cascade activation, the IIIPI3K complex produces PI3P and induces other Atg proteins, such as the Atg12-Atg5-Atg-16 and LC3 (Atg8)-phosphatidylethanolamine complexes. After translation, proLC3 is proteolytically cleaved generating LC3-I. Upon induction of autophagy, LC3-I is conjugated by the Atg7, Atg3 and Atg12-Atg5-Atg16L complexes to the highly lipophilic phosphatidylethanolamine (PE) moiety to generate LC3-II. Finally, PE promotes integration of LC3-II into lipid membranes allowing autophagosomes formation. Due to its crucial role as a natural defense mechanism against inflammatory, infectious and degenerative disorders, the autophagic process must be tightly regulated. Several molecular mechanisms of autophagy regulation have been investigated: microRNAs (miRNAs) stand out, among others [5]. These small noncoding RNAs act as negative regulators of specific target mRNAs expression. One single miRNA can act as a post-transcriptional repressor by binding to partially complementary sequences in the 3′UTR sites of various mRNAs. miRNAs which regulate the autophagic process, predominantly targeting the pathway early stages, are called autophagomiRNAs. In several pathological conditions, e.g., degenerative disorders, autophagomiRNAs have been found to be differentially expressed [6].

**Figure 1:**
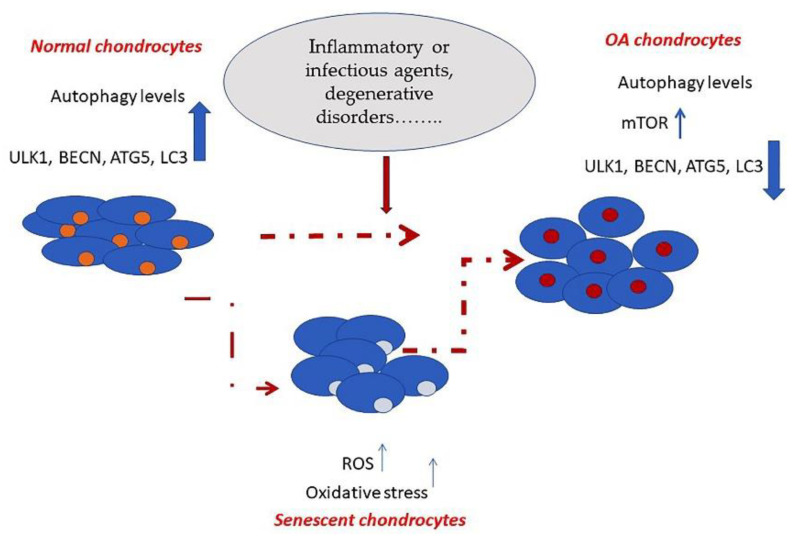
Schematic representation showing changes occurring in the autophagic process in normal versus osteoarthritic chondrocytes.

Increasing evidence suggests that autophagy dysregulation is closely related to the pathogenesis of osteoarthritis (OA) [7] (Figure 1).

OA, a chronic, age-related degenerative disease of articular cartilage, is associated with dramatic changes in cartilage homeostasis, due to an imbalance between degradation and synthesis by chondrocytes. Age-related changes that occur in joints are thought to represent a major risk factor for OA development. OA may develop in any joint, but most commonly, it affects the knee, hip, hand, spine and foot.

Incidence is higher in women than in men, especially beyond age 50. Worldwide estimates indicate that 10% of men and 18% of women ≥ 60 years have symptomatic OA. Disease progression is usually slow but can ultimately lead to joint failure with pain and disability, with considerable socio-economic impact [8].

## 2. Role of the Autophagic Process in Chondrogenic Differentiation

Autophagy is involved in different cellular processes such as the control of epigenetic modifications, metabolic processes, cellular senescence and apoptosis, as well as stem cells differentiation steps [9]. Recent studies have demonstrated that the autophagic process is crucial for stem cell functioning [10,11]. Mesenchymal stem cells have trilineage potency (adipocyte, osteoblast and chondrocyte); they are essential for homeostasis maintenance and tissues repair. Chondrogenesis is a dynamic process associated with morphological changes and metabolic stress [12]. Healthy chondrocytes are essential for a functional cartilage, but their regenerative potential is very limited; hence, both chondrocytic homeostasis and cartilage extracellular matrix integrity are required [11]. Endochondral ossification, a process where chondrocytes differentiate to form the growth plate, is essential in mammalian bone formation. As the growth plate is poorly vascularized, chondrocytes grow in an hypoxic environment [13]. In such a context, autophagy is induced, preventing premature apoptosis of differentiating and mature chondrocytes. In fact, the chondrocytic maturation phase is regulated by mTOR and AMP kinase (AMPK) activity [14]. AMPK, a molecule involved in the regulation of cellular metabolism, acts by inducing autophagy, whereas mTOR, a regulator of cell growth, inhibits autophagy [15]. AMPK and mTORC (complex1 of mTOR) provide an integrated signal by phosphorylating ULK 1 protein kinase in a coordinated way so that the cells are able to respond appropriately to external factors [15]. During endochondral ossification, chondrocytes produce extracellular matrix components: the endoplasmic reticulum (ER), involved in the secretory process, is in a stressful condition. Consequently, ER stress induces autophagy in order to maintain its homeostasis [16]. Recently, it has been demonstrated that a faulty autophagic process induces ER stress and affects chondrogenesis [8]. Under mechanical stimuli, such as compression, autophagy preserves intervertebral disc degeneration. However, a persistent compression stimulus can induce excessive autophagy leading to cellular apoptosis [17]. Ma et al. demonstrated that autophagy is mediated by ROS (reactive oxygen species) in nucleus pulposus cells of rats exposed to compressive stimuli [18]. The levels of reactive oxygen and nitrogen species (RONS) induced by IL-1 can be influenced by oxygen tension. Indeed, environmental oxygen tension in articular chondrocytes has been shown to play an important role in determining their ability to counteract RONS exposure in OA [19]. In addition, we have demonstrated increased expression during physical activity of chondrogenic transcription factors SOX9 in circulating mesenchymal progenitors associated with autophagy [20]. This finding suggests the beneficial role of physical exercise in preserving chondrogenesis. Autophagy adjusts mitochondrial activity in stem cells in order to provide the best metabolic conditions, thus limiting ROS production, preventing metabolic stress and genome damage. Mitochondria, as cellular energy producers, are involved in many vital processes [21]. Mitochondria dysfunctions cause cellular damages in many aging-related diseases such as osteoarthritis [22]. Maintenance of a correct mitochondrial functionality appears therefore very important. Mitophagy, the autophagy process involving damaged mitochondria, contributes to a correct mitochondrial activity [23]. Different mitophagy mechanisms as well as different mitophagy inducers have been investigated. Generally, mitophagy can be PRKN (parkin RBR E3 ubiquitin protein ligase)-dependent or PRKN-independent [24]. In animal models, it has been demonstrated that the chondrogenic commitment involves LC3-Dependent Mitophagy [25] and that mitophagy improves the chondrogenic differentiation potential of Adipose Stem Cells [26]. Additionally, mitophagy regulators such as PINK1, PRKN, BNIP3 and MFN2 were shown to be involved in OA pathogenesis [24]. In particular, the PINK1-PRKN pathway plays an important role in the induction of mitophagy in chondrocytes [24]. Therefore, mitophagy appears as an effective contrast tool against OA development.

## 3. MicroRNAs Involvement in the Autophagy Process

Autophagy dysfunctions are involved in cartilage deterioration, whereas induction of autophagy can counteract cartilage degeneration. ULK1, LC3 and beclin, autophagy-related proteins, are expressed in cartilage. However, their expression is reduced in OA disorder [27]. In addition, the expression of LC3, ULK-1, P62 and Beclin-1 in chondrocytes is downregulated by miR-411 [28]. Recently, it has been demonstrated that miR-375 worsens knee osteoarthritis by targeting the autophagy related protein ATG2B [29]. Reduced circulating miR let-7e levels are associated to increased apoptosis and reduced autophagy in knee OA cartilage [30]. Undoubtedly, miRNAs play a crucial role in cartilage homeostasis as well as in the autophagic process. In particular, level changes in miRNAs targeting the autophagic pathway (autophagomiRNAs) may influence the development of OA [31].

**Figure 2:**
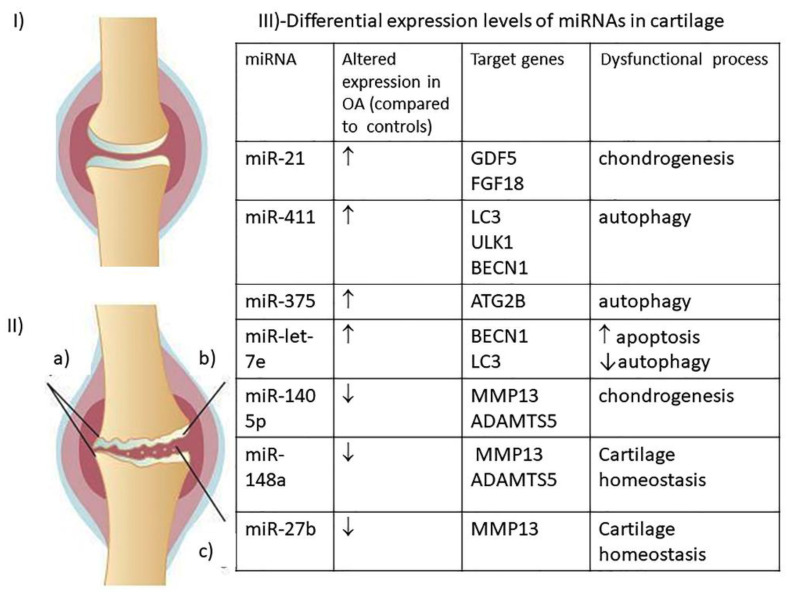
Schematic representation of changes that occur in a healthy knee joint (**I**) upon OA degenerative process (**II**): (a) osteophytes production; (b) cartilage thinning; (c) cartilage fragmentation. (**III**) The table reports a few miRNAs cited within the text, which are differentially expressed in OA cartilage, compared to healthy cartilage. ↑ = enhanced expression; ↓ = lowered expression.

MiRNAs may be recovered from biological samples such as plasma, serum, cartilage and synovial fluid, as they are secreted from cells in exosomes or encapsulated within microvesicles. Quantification and analysis of several autophagomiRNAs reveal differential expression levels in samples from OA patients, compared with healthy controls [4]. Panels of cartilage miRNAs, which appear deregulated in OA, have been proposed as diagnostic/prognostic markers. A sample of those cited in this review is shown in Figure 2.

## 4. Transcription Factors in OA

Transcription factors are DNA-binding proteins which play a central role in regulating gene expression [32] and, consequently, are involved in cell signaling as well as in cellular proliferation and differentiation [33].

Some transcription factors, defined Master regulators, commit progenitor cells differentiation by inducing the expression of lineage-specific genes [34]. Generally, transcription factors recognize highly conserved sequences (6 to 12 bp long) upstream target genes [35]. Notably, the same transcription factors can differently modulate gene expression on the basis of specific interactions [36]. Furthermore, posttranslational modifications, such as phosphorylation/dephosphorylation, may regulate transcription factors efficiency [37]. Transcription factor SOX9 is the master regulator of chondrogenic differentiation. It acts by inducing mesenchymal cells condensation and proliferation and inhibits chondrocyte senescence [38]. Cartilage is absent in Sox9-knockout murine embryonic stem cells; human SOX9 haploinsufficiency induces lethal skeletal malformations [39].

The expression of SOX9 is reduced in chondrocytes of OA patients [40]. In cartilage, it has been shown that SOX9 regulates miR-140 levels in zebrafish and mammalian cells [41], while miR-1247 as well as MiR-30a and miR-145 target SOX9 [42,43,44].

By performing an enrichment analysis, it has been observed that transcription factors, such as activator protein 1 (AP-1), CCAAT-enhancer-binding protein (C/EBP) and the activator of transcription 3 (STAT3), may be involved in the regulation of genes whose expression is altered in OA [45]. A transcriptome study showed altered expression of transcription factors such as JUN, EGR1, JUND, FOSL2, MYC, KLF4, RelA and FOXO in the cartilage of OA human knee [46].

The transcription factors AP-1, runt-related transcription factor 2 (RUNX2), NFkB, HIF2 α and T-cell factor/lymphoid enhancer factor (TCF/LEF) regulate the cartilage ECM-degrading molecules MMP3 and MMP13 (collagenases), whereas ADAMTS4 and ADAMTS5 (aggrecanases) are regulated by the transcription factors NFAT, RUNX2, SOX4, SOX11 and NFkB [47,48]. Interestingly, during chondrogenesis in human adipose-derived stem cells (hADSCs), it has been demonstrated that miR-193b, miR-199a-3p/has-miR-199b-3p, miR-455-3p, miR-210, miR-381 and miR-92a target RUNX2 [49].

The upregulation of SOX4 and SOX11 in mouse cartilage is associated to impaired cartilage and increased expression of ADAMTS5 and MMP13 [48]; chondrocyte proteins ACAN and COL2A1 are regulated by the transcription factors SOX5, SOX6 and SOX9 [50]. During cartilage formation, miR-193b targets SOX4 and miR-455-3p targets SOX-4, SOX5, SOX6 and SOX9 [49]. 

Transcription factor EB (TFEB) and the zinc-finger protein with KRAB and SCAN domains 3 (ZKSCAN3) are important master regulators of autophagy. TFEB, known to induce autophagy in HeLa cells [51], is reduced in a OA mouse model and in OA human knee cartilage [52]. On the contrary, ZKSCAN3 inhibits autophagy by blocking the expression of ULK1 and LC3 genes [53]. ZKSCAN3 expression has not been evaluated in chondrocytes; however, it has been demonstrated that ZKSCAN3 knockout induces premature aging in Mesenchymal Stem Cells [54]. SIRT1, whose levels are increased in early chondrocytes but are reduced in severe OA, promotes autophagy by acting on FOXO family transcription factors [55]. FOXO1 and FOXO3 induce autophagy [56]. In fact, ATG genes expression is reduced in chondrocytes under oxidative stress conditions due to FOXO1 or FOXO1/FOXO3 knockdown [57], while a constitutively expressed mutant FOXO1 increased the expression of LC3 and Beclin in normal chondrocytes [58]. Interestingly, in osteoarthritis samples, transcription factors FOXO are reduced [46] and the activated serine/threonine kinase AKT, which phosphorylates the FOXO transcription factors, is higher in OA cartilage compared to normal cartilage [59]. By performing in silico analysis and also in vitro and in vivo experiments, it has been demonstrated that during skeletogenesis FOXO1 is targeted by miR-182 [60]; bioinformatic analysis has shown that miR182 plays a critical role in OA [61].

## 5. In Vitro and In Vivo Models for OA Studies

Different models have been employed to investigate OA pathogenesis. In vitro systems have been established by using human or murine primary cultures and cell lines. Usually, a mechanical load or inflammatory cytokines are applied to cells in order to mimic OA conditions. In particular, OA mimic conditions are applied to cells growing in a monolayer, in a scaffold or in a co-culture system [62]. The use of 3D cell models may represent a good alternative to 2D cultures. 3D models for the in vitro analysis of subchondral bone and articular cartilage currently exist in a variety of forms, including explants and scaffold-based or scaffold-free systems, each of which has its own advantages and disadvantages. 3D systems make it possible to observe the various cellular interactions and to evaluate any changes due to the addition of therapeutic molecules [63]. However, investigations of explanted tissues allow assessment of the extracellular matrix and cellular interactions to recapitulate in vivo alterations [64].

In vivo models provide the possibility to evaluate pain, cartilage degeneration and the bone remodeling process. OA in animal models can be induced or spontaneous. In particular, OA can be induced surgically or chemically; induced models can also be generated genetically [65]. Animal models with naturally occurring OA, such as aged animals, can also be used.

Usual animal models for OA are: mouse, rat, Syrian hamster, rabbit, horse and also cat and dog [65]. Zebrafish has also been employed for the study of OA. The zebrafish model, due to its ease of being genetically manipulated and its rapid development, appears to be very useful. For example, a COL10A1 knockout zebrafish model has been generated for the study of OA [66].

In fact, the craniofacial cartilage of zebrafish larvae is as mechanically sensitive as the human one [67]. Different models for the zebrafish jaw development are available, including wild-type fish [68] and mutants [68]. In addition, zebrafish larvae in different gravitational fields have been used [68]. The zebrafish model is also suitable for studying miR-mediated joint degeneration. In particular, zebrafish dicer1 mutant shows impaired craniofacial development and overexpression of SOX10 [69]. SOX9 controls miR140 and miR-29; miR92a regulates BMP signaling in zebrafish cartilage [41,70].

The introduction of the CRISPR/Cas9 technology in recent years has further expanded the possibilities of originating cellular and animal models to study the role of different genes and regulatory factors involved in degeneration, regeneration and inflammatory processes associated with OA. The CRISPR/Cas9 system in fact not only allows to knock out specific genes and functions, but by using modified versions of the Cas9 enzyme, it becomes possible to originate recombinant proteins that can act as transcriptional activators and repressors, as well as epigenetic modulators [71], to study in a more precise way the regulation of specific genes [72]. The possibility to originate new cellular and animal models [73,74] will be very helpful to overcome the limited availability of animal models of the disease. In addition, the availability of different models will make drug screening aimed at identifying new therapies much more efficient, as will the possibility of studying new therapeutic approaches based on gene therapy [75].

Various treatments have been developed to counteract or, at least delay, OA clinical progression. Anti-inflammatory drugs are employed, as well as non-pharmacological treatments, such as electromagnetic stimulation, shock wave therapy and biomechanical intervention [76]. Surgical treatment (e.g., total joint replacement) is chosen for advanced OA.

## 6. Therapeutic Targets in OA

### 6.1. Autophagy

Considering its prominent role in cell pathophysiology, autophagy can be therapeutically targeted and modulated at various points of its pathways in human diseases [77]. Preventing autophagy inhibition and decreasing ROS production are strategies with therapeutic potential against OA. mTOR, a signaling molecule in the autophagy pathway, has been chosen as a target in experimental studies. Rapamycin, an mTOR inhibitor, has been proven to delay cartilage degeneration upon intra-articular injection in a murine OA model [78]. Isoimperatorin and glucosamine can ameliorate osteoarthritis by activating autophagy and inhibiting mTOR pathway [79]. Resveratrol (RSV) can activate Sirtuin 1 (SIRT1), an autophagy promoter, thus inhibiting OA progression [80].

### 6.2. Inflammation

The existence of an important inflammatory component in OA is well known. Damage-associated molecular patterns (DAMPs) and various sources of oxidative stress contribute to inflammation [81]. An in vitro screening for DMOADs (disease-modifying osteoarthritis drugs) revealed the strong chondrogenic/chondroprotective effects of BNTA (*N*-[2 bromo-4-phenylsulfonyl-3-thienyl]-2-chlorobenzamide) [82]. BNTA beneficial effects may be ascribed to its induction of SOD3 expression and superoxide anions elimination. Resveratrol (RSV), already cited, is a powerful antioxidant as well.

The condroprotective role of Resveratrol (RSV) may also be associated to its ability to inhibit inflammation and the NF-κB signaling pathway. The activation of transcription factor NF-kB, an essential mediator of inflammatory responses, depends on the inducible degradation of its inhibitor, IkBα [83]. In an in vitro model (IL-β1 treated human chondrocytes), the inflammatory response was significantly inhibited by RSV administration [84]. Molecular evidence demonstrated that RSV relieved the inflammatory response by inhibiting IkBα degradation. It is worth recalling that baseline NF-kB activity plays a positive role in healthy cartilage, ensuring chondrocytes differentiation and survival. Environmental and inflammatory cues exacerbate NF-κB response, which leads to the expression of matrix metalloproteinases (MMPs), cyclooxygenases (COX) and inflammatory cytokines (e.g., IL-1, IL-6, IL-8 and TNF). The MMPs family includes several members, which are secreted as inactive pro-forms. Once the pro-domain is cleaved, the active enzymes proceed to ECM proteins degradation. They are involved in physiological processes, such as embryonic development and tissue remodeling and are overexpressed in degenerative processes such as OA. MMP13, also called collagenase 3, is a major enzyme targeting cartilage for degradation. It targets type II collagen, but it also degrades proteoglycans, type IV and type IX collagen, osteonectin and perlecan. MMP13 overexpression is typically observed in OA patients [85,86]. The ADAMTS (a disintegrin and metalloproteinase with thrombospondin motifs) family of aggrecanases also contributes to proteoglycan/aggrecan depletion. ADAMTS 4 and 5 have been identified as the main aggrecanases involved in OA development [87]. The above-mentioned catabolic enzymes play an important role in OA progression, and therefore, represent interesting therapeutic targets for articular cartilage degradation slowdown [88,89,90,91].

### 6.3. Cell Senescence

**Figure 3:**
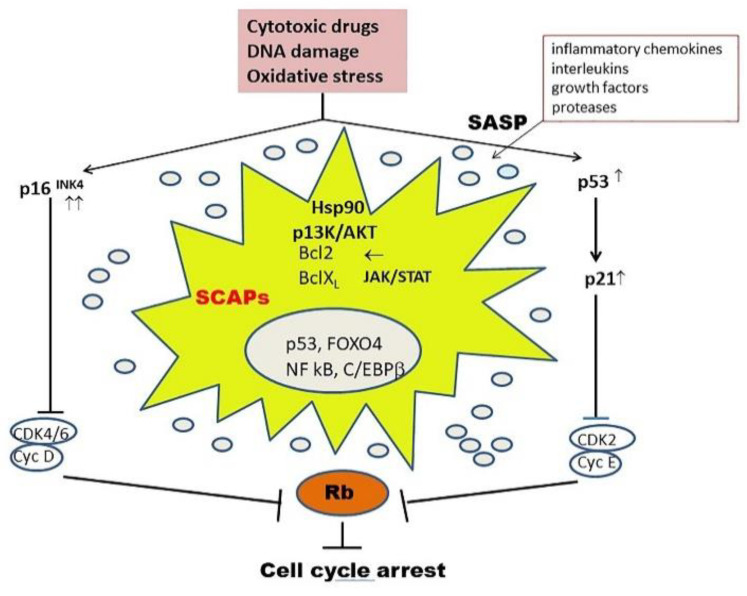
Cellular senescence (i.e., permanent cessation of cell division) is triggered by a variety of stresses. The tumor suppressor p16 ^INK4^, as well as the p53 and p21 proteins, function as cyclin-dependent kinase inhibitors, preventing phosphorylation of the Rb protein and consequently arresting the cell cycle. They release SASP factors which severely damage neighboring cells. Senescent cells can escape apoptosis by upregulation of pro-survival pathways (SCAPs = senescent cell antiapoptotic pathways). Senolytic therapies primarily target SCAPs, as exemplified in Table 1.

Cell senescence is a stress-activated molecular program that prevents damaged cells from further proliferation. Autophagy and cellular senescence share several stimuli (e.g., damaged organelles or macromolecules, oxidative stress). Although autophagy is generally considered to suppress cellular senescence, various studies have suggested that it may also promote it [92,93]. Senescent cells (SC) accumulate in chronic age-associated diseases, such as OA. Their inflammatory senescence-associated secretory phenotype (SASP) severely damages neighboring cells. SC appear to be resistant to apoptosis due to the upregulation of pro-survival pathways related to P13K/AKT, p53-p21 and antiapoptotic BCL family members, among others (Figure 3).

On the basis of the above observations, recently, researchers have started testing molecules known to target pro-survival pathways [94]. Molecules meeting these requirements are called senolytics, as they selectively induce SC apoptosis [95]. Table 1 reports a few examples of senolytics and their targets. Targeting is achieved by nanoparticle-based delivery of senolytics [96].

To our knowledge, there are two current clinical trials in the US involving knee OA patients treated with senolytic regimens:

- UBX0101(NCT04129944).
- Fisetin (NCT04210986).

### 6.4. microRNAs

Other molecular targets for therapeutic options may be specific miRNAs, whose dysregulation plays an important role in OA. AntagomiRNAs and/or miRNA mimics may be synthesized and delivered into experimental models in order to remodel microRNAs levels. In detail, antagomiRNAs are synthetic oligonucleotides which can inhibit specific endogenous miRNAs by base-pairing, hence hindering miRNA-target mRNAs matching. MiRNA mimics instead act in the opposite way: they can be introduced by transient transfection to enhance the regulatory action of endogenous identical miRNAs. Their therapeutic effectiveness depends on the actual possibility to deliver them to the cartilaginous tissue.

OA animal models (mice and rats) have been employed in promising studies so far. Upregulation of miR21, for example, is associated with OA in humans (Figure 1III) and it has also been observed in experimental OA murine models [97]. Intra-articular injection of miR-21 mimics caused a significant worsening of cartilage degradation, whereas antagomiR-21 injection had the opposite effect. Another experiment on similar OA rat models [98] demonstrated the therapeutic efficacy of miR-140-5p, an autophagy regulator (Figure 1III). Rats were treated with intra-articular injection of human umbilical cord stem cells (hUC-MSCs) ± miR-140-5p mimic. The authors demonstrated that hUC-MSCs+ miR-140-5p mimic differentiated to chondrocytes and induced rat’s cartilage repair much more efficiently than hUC-MSCs.

## 7. Novel Therapeutic Strategies

In addition to the above clinical trials with senolytic molecules, other experimental treatments are based on the use of miRNAs. An efficient way to deliver therapeutic miRNAs involves MSC-derived extracellular vesicles (ECVs) [99] ECVs (100–1000 nm diameter) while exosomes (30–100 nm diameter) are released by several cell types; they are delimited by phospholipid bilayer membranes and carry various cellular components, including miRNAs, which—in this way—are protected from degradation. Surface CD markers allow tracking ECVs’ origin while adhesion molecules facilitate internalization by recipient cells. Studies conducted on animal models or in in vitro human models (e.g., Il-1β treated synovial fibroblasts) demonstrated that hMSCs derived exosomes may promote chondrocytes proliferation and cartilage repair [100,101].

Exosomes may also be exploited as therapeutic agents because of their targeting capacity and loading ability. By simple incubation, they can be loaded with hydrophobic molecules, such as curcumin and other antioxidants, ensuring considerable stability and bioavailability [102]. Tissue engineering strategies are under development in order to ensure EVs maintenance within the damaged cartilage [103]. Various scaffold types have been tested. Among others, hyaluronic acid (HA) and collagen hydrogels seemed eligible biomaterials for cartilage regeneration. Liu et al. [104] successfully incorporated EVs obtained from iPSC-MSC into a hydrogel glue, which was then implanted into a rabbit articular defect model. The evolution of 3D printing technology offers new chances to exosome-based tissue engineering strategies. Chen et al. tested a 3D printed cartilage ECM/gelatin methacrylate (GeMA)/exosome scaffold, which restored chondrocyte mitochondrial dysfunction and enhanced chondrocyte migration, facilitating cartilage regeneration in an OA rabbit model. Interestingly, the 3D-printed scaffold could retain exosomes for 14 days in vitro and for ≥7 days in vivo [105].

Innovative therapeutic approaches based on genome editing using the CRISPR/Cas9 technique are also being evaluated. The technique has a high potential in regenerative medicine and cell-based applications for cartilage repair [106].

A first therapeutic approach is based on exogenous-cell-based therapy, by delivering chondrocytes or MSCs previously engineered in vitro. Using this approach, Seidl et al. reported that by targeting the MMP13 gene, increased accumulation of cartilage matrix protein type II collagen was achieved using edited cells [107], while the in vitro knockout of IL1-R1 in chondrocytes before injection reduced inflammation, improving cell-therapy results [108]. Other potential therapeutic target genes which have been investigated are osteocalcin [109] and hyaluronan synthase 2 (HAS2) [110].

An alternative approach relies on intra-articular injection of adeno-associated viral vectors expressing CRISPR/Cas9 components to target MMP13, IL-1β and NGF; Zhao et al. [89] reported that the inactivation of these genes may be useful for both pain management and joint maintenance. Moreover, the Cas9 enzyme may be suitably engineered to originate fusion products with factors such as activators and epigenetic modifiers: activation or repression of genes involved in inflammation could have an important therapeutic potential in OA. Epigenetic editing may also allow programming genes networks to target stem cell differentiation for their clinical employment for regenerative therapy [111].

Delivery systems involving non-viral vectors may be preferable in order to avoid inflammatory responses in joints, which can cause adverse side effects [112]. Studies aimed at evaluating different viral and non-viral vectors for the efficient delivery of the CRISPR/Cas9 system at the joint level are needed before this new technology can be proficiently translated into the clinic.

## 8. Conclusions and Perspectives

**Figure 4:**
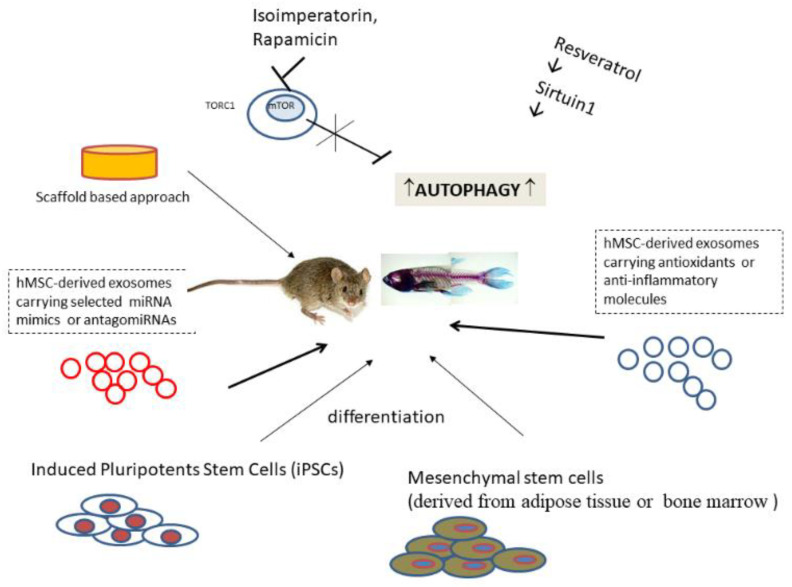
Therapeutic strategies successfully tested in animal OA models (see text).

As the human lifespan is progressively expanding, the incidence of degenerative disorders associated with ageing, such as OA, is increasing. Traditional treatment options for OA aim at relieving symptoms (pain, inflammation) and at delaying severe disability in patients. Cell-based therapies focusing on the restoration of damaged articular cartilage have been tested on experimental animal models mimicking the OA phenotype (Figure 4).

MicroRNAs involved in the regulation of articular cartilage homeostasis, autophagy and apoptosis show differential expression in OA, i.e., they are either upregulated or downregulated in patients compared to healthy controls. MicroRNAs, which can be recovered from cartilage, blood and synovial fluid, may, therefore, represent diagnostic/prognostic biomarkers. A special attention has been paid to extracellular vesicles-associated miRNAs: they represent the most reliable biomarkers, as they are protected from degradation. Most ECV/exosomes recovered from the OA microenvironment are disease detectors, while ECVs/exosomes released by MSCs (cartilage progenitor cells) have been shown to exert therapeutic effects on cartilage tissue in experimental models. Bioscaffolds loaded with therapeutic exosomes might be safer and more effective (due to the gradual release) than repeated intra-articular injections. In the future, 3D printed scaffolds might also allow the design of personalized and precision treatments [113]. Cellular senescence, which burdens the OA phenotype, also represents an emerging opportunity for novel therapeutic approaches through the exploitation of senolytics.

## Figures and Tables

**Table 1:** Examples of Senolytics with Their Respective Targets.

Senolytic Drug	Targeted Molecules/Pathway
FISETIN	⊥ P13/AKT/mTOR ⊥ Bcl2-xL
QUERCETIN	⊥ P13/AKT/mTOR ⊥ Bcl2-w
NAVITOCLAX	⊥ Bcl2, Bcl-xL, Bcl-w
FOXO4-DRI	⊗ FOXO4-p53 interaction, no p53 in the nucleus
USP7 inhibitor	MDM2 ubiquitination
UBX0101	MDM2, p32

## Data Availability

Not applicable.
